# Pembrolizumab-Induced Pancreatic Exocrine Insufficiency Complicated by Severe Hepatic Steatosis

**DOI:** 10.7759/cureus.26596

**Published:** 2022-07-05

**Authors:** Alex S Hong, Naveed Sarwar, Robert D Goldin, Ameet Dhar, Lucia A Possamai

**Affiliations:** 1 Gastroenterology and Hepatology, Imperial College School of Medicine, London, GBR; 2 Medical Oncology, Department of Oncology, Imperial College Healthcare NHS Trust, Charing Cross Hospital, London, GBR; 3 Section for Pathology, Department of Metabolism, Digestion and Reproduction, Imperial College London, London, GBR; 4 Gastroenterology and Hepatology, Department of Hepatology, Imperial College Healthcare NHS Trust, St. Mary’s Hospital, London, GBR

**Keywords:** immunotherapy, immune-related adverse event, hepatic steatosis, pancreatic exocrine insufficiency, pembrolizumab

## Abstract

Anti-programmed death receptor-1 (anti-PD-1) monoclonal antibodies (mAbs) are used to treat an increasing range of cancers. However, the distinct toxicity profile of immune-related adverse events (irAEs) is a frequent drawback of their clinical application. Among the more common irAEs are hepatitis and colitis, which are diagnosed and graded in patients based on elevated serum liver enzyme levels and increased stool frequency, respectively, and both of which often require treatment with high-dose corticosteroids. Herein, we describe the case of a patient who developed severe transaminase elevation and diarrhoea due to an unusual irAE, which was successfully treated without corticosteroids.

## Introduction

Anti-programmed death receptor-1 (anti-PD-1) monoclonal antibodies (mAbs), such as pembrolizumab, are members of a more general group of immunotherapeutic drugs known as immune checkpoint inhibitors (ICIs), which have demonstrated promising therapeutic outcomes in various malignancies [1]. Anti-PD-1 mAbs antagonise the interaction between the programmed death-1 receptor (PD-1), which is largely but not exclusively expressed on T cells, and the programmed death ligand-1. The direct blockade of PD-1 results in the attenuation of the inhibitory effect of these receptors on T-cell effector mechanisms, thus augmenting the immune response against tumour cells [1]. However, the ability of anti-PD-1 mAbs to induce potent anti-tumour activity through the enhanced T cell-specific immune response has also been implicated in the development of non-specific immune activation, resulting in various systemic and organ-specific immune-related adverse events (irAEs) [2].

The gastrointestinal tract is one of the commonest sites of irAEs associated with immunotherapy. With single-agent ICI treatment, 5-10% of patients will develop hepatitis, and in 1-2% of patients, this will be a severe, grade 3/4 reaction [3]. Initially, irAE hepatitis was thought to mimic autoimmune hepatitis. However, with greater experience, it is clear that it is a distinct clinical and pathological entity [4]. Histologically, it is characterised by lobular hepatitis with spotty or confluent necrosis and a predominance of T cells [5]. In the context of anti-cytotoxic T-lymphocyte-associated protein-4 (anti-CTLA-4) treatment, but not anti-PD-1 monotherapy, hepatic granulomas may be seen [5]. According to the European Society for Medical Oncology (ESMO) guidelines, irAE hepatitis is classified by elevation in serum liver enzyme levels, predominantly alanine transaminase (ALT) and aspartate transaminase (AST) [3]. These guidelines recommend cessation of ICI treatment, combined with the initiation of intravenous (IV) methylprednisolone (2 mg/kg) for patients with an ALT > 400 IU/L. The American Society of Clinical Oncology guidelines have similar treatment thresholds, also recommending high-dose parenteral corticosteroids in patients with grade 3 hepatitis [6].

Colitis is also a relatively common irAE observed following treatment with ICIs. IrAE colitis is strongly associated with anti-CTLA-4 treatment, with 8-22% of patients developing any grade of irAE colitis [3]. Anti-PD-1 monotherapy may also be complicated by the development of immune-related colitis. Treatment protocols use the frequency of bowel opening, along with other symptoms to grade irAE colitis and stratify risk and treatment recommendations. As with irAE hepatitis, corticosteroids are used to treat irAE colitis. However, in severe cases, anti-tumour necrosis factor-alpha agents, such as infliximab, may be used [3,6].

As treatment experience with ICIs grows, the emergence of rare and novel irAEs continues. Isolated pancreatic exocrine insufficiency (PEI) resulting from anti-PD-1 treatment has been described in some reports as a rare gastrointestinal irAE [1,2,7-10]. In the surgical context, PEI has been implicated in the onset of non-alcoholic fatty liver disease (NAFLD) and, or non-alcoholic steatohepatitis (NASH) following surgery involving various types of pancreatic resection [11-19]. Interestingly, the development of NASH in a non-surgical patient with a background devoid of obesity has been reported in the literature, again where PEI was suspected to be the precipitant [20]. The culmination of these findings reflects our current lack of understanding of the unusual causes of hepatic steatosis, and there is growing evidence to support the role of PEI as a precipitating factor in the development of these hepatic syndromes. Therefore, the causes of PEI may serve as an indirect trigger for NASH and NAFLD.

Here, we describe a case in which PEI secondary to pembrolizumab treatment masqueraded as the more common irAEs of colitis and hepatitis.

This article was previously presented as a poster presentation at the United European Gastroenterology (UEG) Week 2021 Virtual on October 4th, 2021.

## Case presentation

A 62-year-old female patient with a past medical history of hypothyroidism and appendicectomy presented to her general practitioner complaining of fatigue in August 2017. An elevated gamma-glutamyl transferase (γGT) was noted on routine blood tests. An abdominal ultrasound scan (USS) was performed, during which a right-sided renal mass was incidentally found. A staging computerised tomography (CT) scan of the thorax, abdomen, and pelvis also identified a renal mass with no evidence of metastatic disease. The renal lesion was biopsied and histological analysis confirmed a diagnosis of clear cell renal carcinoma. The patient subsequently underwent a right-sided open radical nephrectomy. She was treated with adjuvant pembrolizumab, with cycle one initiated in November 2017.

During treatment, the patient complained of generalised malaise, accompanied by night sweats, non-productive cough, and nasal congestion in April 2018. As a result of the following symptomatology, treatment was withheld at the patient’s request in April but was recommenced in mid-May 2018. However, at a follow-up appointment in June 2018, the patient described a few weeks’ history of diarrhoea with bowels opening five-to-seven times per day and was subsequently diagnosed with a grade 2 irAE colitis on this basis (Table 1). Prednisolone was administered to treat the colitis according to ESMO treatment guidelines [3]. Unfortunately, the patient’s symptoms did not improve. The patient then developed steroid-induced diabetes, and the prednisolone was eventually tapered down and stopped. The patient ultimately withdrew from the pembrolizumab therapy at her own discretion after a total of nine cycles.

**Table 1:** ESMO guidelines for the assessment of the severity of post-ICI hepatitis, colitis and diarrhoea. The severity of hepatitis is graded according to the elevation of ALT or AST in relation to the upper limit of normal (ULN). In contrast, the severity of colitis is graded according to the frequency of liquid stools per day in combination with symptomatic findings. (Adapted from Haanen et al. [3]). ESMO: European Society for Medical Oncology; ICI: immune checkpoint inhibitor; irAE: immune-related adverse event; ALT: alanine transaminase; AST: aspartate transaminase; and ULN: upper limit of normal.

irAE grade	Hepatitis	Colitis and diarrhoea
Grade 1	ALT or AST > ULN-3x ULN	< 4 Liquid stools per day over baseline, feeling well
Grade 2	ALT or AST, 3-5x ULN	Four-to-six liquid stools per day over baseline, or abdominal pain, or blood in stool, or nausea, or nocturnal episodes
Grade 3	ALT or AST, 5-20x ULN	≥ 7 Liquid stools per day or life-threatening
Grade 4	ALT or AST > 20x ULN	

In December 2018, during a routine screening visit, the patient described ongoing diarrhoea for the past six months, associated with new-onset faecal incontinence. The stool was described as loose, pale, and foul-smelling, accompanied by an orange oily residue. The patient denied the presence of any blood in the stool. Additionally, the patient complained of unintentional weight loss from 81 to 66 kg (19% reduction in body mass) over six months from June to December 2018 with progressively worsening fatigue.

Biochemical investigations revealed a largely but markedly deranged set of liver function test (LFT) results, including elevated levels of ALT, alkaline phosphatase (ALP), and γGT, accompanied by an initial mildly prolonged prothrombin time of 18.1 seconds, which was corrected to 14.8 seconds following administration of a single dose of IV vitamin K (Table 2). The satisfactory response to vitamin K, in hand with the suboptimal level of vitamin D, was reflective of a degree of fat-soluble vitamin deficiency. The thyroid function-related biochemistry, which was suggestive of subclinical hypothyroidism, was likely related to the patient’s history of hypothyroidism. Otherwise, further serological testing revealed normal immunoglobulins, including normal immunoglobulin G subclass antibodies and negative antinuclear antibodies and autoantibodies. An extensive virology panel excluded infection with cytomegalovirus, Epstein-Barr virus, human immunodeficiency virus, and hepatitis viruses.

**Table 2:** Summary of the findings from the biochemical investigations. ALT: alanine transaminase; ALP: alkaline phosphatase; and γGT: gamma-glutamyl transferase.

Biochemical investigation	Patient’s result	Normal reference range	Interpretation
ALT (units/L)	459	0-34	High
ALP (units/L)	298	30-130	High
γGT (units/L)	232	< 40	High
Bilirubin (µmol/L)	9	0-21	Normal
Prothrombin time (seconds)	18.1	13-16	High
Vitamin D (nmol)	37.2	> 50	Low
Thyroid stimulating hormone (milliunit/L)	5.82	0.30-4.20	High
Free thyroxine (pmol/L)	12.9	0.90-23.0	Normal

Based on these symptoms and laboratory findings, irAE hepatitis and colitis secondary to pembrolizumab were initially suspected. Additional investigations were requested, and the patient was referred for a specialist Hepatology opinion and review.

**Figure 1:**
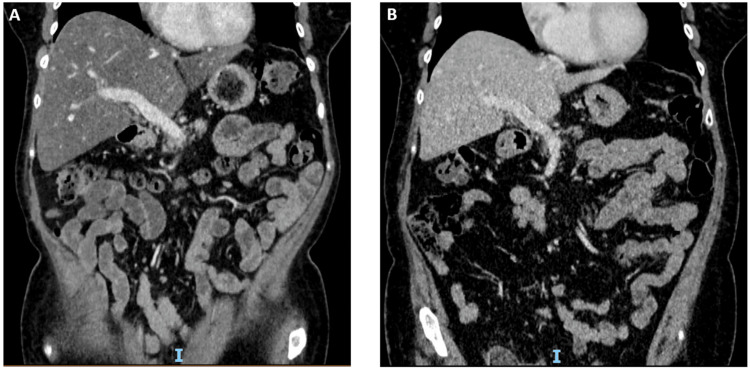
Abdominal CT scan performed in January 2019 (A) and October 2019 (B), respectively. (A) The liver demonstrates a homogenous reduction in density, in keeping with hepatic steatosis. No focal liver lesion is demonstrated on this background. (B) Resolution of hepatic steatosis and an increase in subcutaneous fat following successful treatment of PEI. CT: computerised tomography; PEI: pancreatic exocrine insufficiency.

Further investigations revealed undetectable levels of faecal elastase. A liver USS demonstrated a normal-sized liver, smooth in contour, showing a diffuse heterogeneous increase in echotexture. A simple cyst measuring 10 mm was identified in the right lobe. Focal lesions were not seen, and there was no evidence of intrahepatic duct dilatation. The main portal vein was patent with normal hepatopetal flow. The common bile duct was of normal calibre, measuring 4 mm. The gallbladder was well-distended, thin-walled, and contained multiple mobile calculi. A CT scan of the abdomen (Figure 1A) demonstrated a reduced density of the liver, in keeping with fatty infiltration. The pancreas appeared normal on CT imaging.

The initial FibroScan® (Echosens, Paris, France) liver stiffness measurement (LSM) and controlled attenuation parameter (CAP) values were 4.7 kPa (normal range, 2-7 kPa) and 312 dB/m, respectively (Table 3), suggesting no significant liver fibrosis but severe hepatic steatosis (grade 3, severe hepatic steatosis, defined as CAP > 280 dB/m).

**Table 3:** FibroScan® measurement at three different time points in our patient. The LSM values were consistently found to be within the normal range, suggesting an absence of fibrotic change within the liver. However, the CAP value was initially significantly raised, indicating severe hepatic steatosis (> 280 dB/m). The CAP value diminished in the most recent FibroScan® recording, following pancreatic enzyme replacement therapy (PERT). LSM: liver stiffness measurement; CAP: controlled attenuation parameter.

Date	LSM (kPa)	CAP (dB/m)
January 9th, 2019	4.7	312
February 20th, 2019	4.7	320
September 4th, 2019	3.8	253

**Figure 2:**
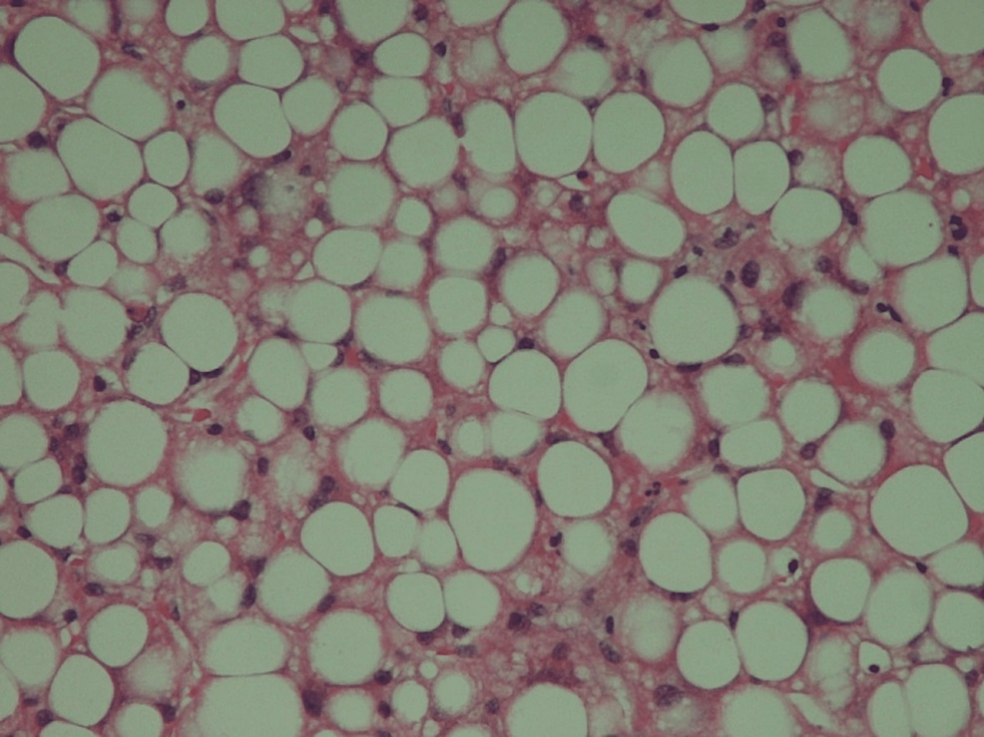
Liver biopsy findings. The results of the liver biopsy revealed a bland, but diffuse and severe steatosis, without features of an immunotherapy-related hepatitis or of fibrosis.

Further imaging with magnetic resonance cholangiopancreatography was unremarkable. A colonoscopy study revealed normal mucosal appearances with randomised colonic biopsies revealing a patchy increase in eosinophils but no significant inflammation. A liver biopsy was also performed, which demonstrated diffuse, severe steatosis without evidence of fibrosis or immune-mediated hepatitis (Figure 2).

Based on the symptoms of steatorrhoea with undetectable faecal elastase levels, fat-soluble vitamin deficiencies, and weight loss, a diagnosis of pembrolizumab-induced PEI was established. The hepatic steatosis was thought to be secondary to PEI rather than a direct irAE. The patient was treated with pancreatic enzyme replacement therapy (PERT) with 40,000U Creon with meals and 10,000U with snacks. The dose of the supplement required up-titration to 70,000U with meals and 20,000U with snacks to achieve a full symptomatic response. Multivitamins were also prescribed. Given the duration of her symptoms, an established PEI, and previous steroid-associated complications, the decision to refrain from further corticosteroid use was made. The diarrhoea and faecal incontinence resolved completely after the commencement of PERT. The patient’s ALT level fell following the commencement of PERT, lowering to 36 IU/L in September 2019. The patient regained body weight and reported significant improvement in her fatigue. The most recent CT scan in October 2019 (Figure 1B) revealed no recurrence of the clear cell renal carcinoma, and her pancreas was described as atrophic, but the liver fat content had normalised. A repeat FibroScan® after a few months of PERT demonstrated an improvement in the CAP score to 253 dB/m (grade 1, mild hepatic steatosis, defined as CAP in the range of 248-268 dB/m) (Table 3), which was compatible with the resolution of the patient’s hepatic steatosis in line with the CT and biochemical findings.

## Discussion

Anti-PD-1 mAb therapy is often clinically complicated by irAEs, and their multi-system toxicity profile has been extensively reported in the literature [2]. In our patient, an irAE colitis was initially suspected as the cause when she first developed diarrhoea and faecal incontinence; thus, steroids were appropriately initiated to manage these symptoms as per standard protocol. However, it became apparent that the underlying pathology was not colitis-related, as the patient failed to improve in response to the treatment. The absence of macroscopic or microscopic colitis was later confirmed through colonoscopy and colonic biopsy.

When the patient further developed derangement in her LFTs with a marked elevation in the ALT, the relatively common irAE hepatitis was considered (Table 1). According to current treatment guidelines, this degree of LFT derangement with suspected irAE hepatitis should be treated with 2 mg/kg of IV methylprednisolone [3]. However, given the patient’s very clear description of steatorrhoea with profound weight loss, undetectable faecal elastase levels, and initial imaging suggesting marked fatty infiltration of the liver, an alternative pathology was suspected. Steroid treatment was therefore deferred, and a liver biopsy was performed, revealing significant hepatic steatosis. A diagnosis of hepatic steatosis secondary to PEI was therefore made. Appropriate enzyme supplementation and vitamin replacement were initiated, leading to complete resolution of the patient’s symptoms and reversal of the biochemical and imaging evidence of hepatic steatosis.

There is no clear guidance in the literature on whether irAE PEI should be treated with steroids with the aim of reducing pancreatic inflammation and salvaging any remaining exocrine function. In the case of the more common irAE endocrinopathies presenting with thyroid insufficiency, thyroxine replacement without corticosteroid therapy is recommended. In our patient, the risks of steroid treatment were decided to outweigh the potential benefits, and thus, steroids were withheld. With an undetectable faecal elastase level and months of ongoing symptoms, it was felt unlikely that any significant degree of pancreatic exocrine function remained. The patient had also previously experienced steroid-induced diabetes and was keen to avoid a recurrence.

Whilst several causes of PEI are known, the manifestation of PEI as an irAE associated with anti-PD-1 mAb therapy is uncommon, as illustrated by the paucity of cases documented in the literature [1,2,7-10]. In contrast to some of these reports, our patient did not exhibit any signs of pancreatitis, and CT imaging only revealed features of pancreatic atrophy months after her symptoms started. There was evidence on imaging and biopsy to suggest that our patient had developed severe hepatic steatosis, which, given the timing and absence of other overt risk factors for conventional NAFLD and NASH, was most likely to have evolved from the PEI. The likelihood of this mechanism of pathogenesis was further supported by the onset of the deranged LFTs. Our patient had a normal ALT at her baseline weight, and ALT only became significantly elevated once she had established rapid weight loss. Hence, the chronology of these events was strongly suggestive of the manifestation of non-conventional NAFLD.

Conventional NAFLD and NASH are commonly associated with chronic over-nutrition, resulting in the accumulation of visceral fat and obesity, in the context of hyperlipidaemia and insulin resistance [17]. However, the occurrence of non-conventional NAFLD and NASH, in the setting of post-pancreatic resection, for instance, appears to be significantly associated with PEI, a factor that is postulated to play a crucial mediating role in the pathogenesis of these hepatic syndromes [13,15,17]. The putative mechanism suggests that the post-surgical reduction in pancreatic parenchymal mass may lead to inadequate exocrine function [18], resulting in PEI-induced malnutrition or malabsorption of essential nutrients, further triggering the development of NAFLD and NASH downstream [12,13].

The clinical features of patients with NAFLD and NASH in the setting of post-pancreaticoduodenectomy (PD) were found to be similar to rodent models with methionine- and choline-deficient diet-induced NASH, suggesting that the malabsorption of essential amino acids and, or fat-soluble nutrients such as choline after PD is likely to have a significant degree of involvement in the molecular pathogenesis [17]. Indeed, the malabsorption of certain amino acids has been associated with decreased hepatic lipoprotein synthesis, resulting in the accumulation of fatty acids in the hepatocytes and overall fat deposition in the liver [12,18]. With these progressive metabolic changes, hepatocellular inflammation and damage ensues, leading to fibrosis, cirrhosis, and ultimately hepatocellular carcinoma if left untreated [13,17,18].

It is important to note that malnutrition of other dietary components in patients with PEI or post-PD may also be attributable to the development of hepatic steatosis, as evidence of increased levels of serum taurine and decreased methionine, tyrosine, albumin, cholinesterase, zinc, and total serum cholesterol levels was reported in such patients [13]. The major imbalance of various nutrients indicates a complex mechanism associated with PEI-induced hepatic steatosis, which is far from fully understood.

Whilst a single case of NASH caused by PEI unrelated to obesity or surgery has been reported in the past [20], to our knowledge, this is the first case of hepatic steatosis secondary to PEI, specifically induced by pembrolizumab. As such, it is uncertain whether the mechanism proposed within the surgical context applies to the obesity-independent or immunotherapy-induced setting. Further research is warranted to elucidate the precise mechanism underlying hepatic steatosis caused by PEI in patients receiving pembrolizumab.

## Conclusions

Our case contributes to the growing body of clinical evidence supporting the existence of a wide spectrum of unusual and unexpected irAEs and their associated complications with ICI therapy. Additionally, our case highlights the importance of considering broad differential diagnoses for diarrhoea and LFT derangement in patients receiving ICIs, as immune-related colitis and hepatitis may not always be the cause for the clinical presentation. Early recognition of patients with irAEs is necessary to rule out alternative pathology and, in doing so, hasten the provision of the most appropriate management to minimise the risk of complications and prevent potential mortality. Specifically, PEI should be suspected in cases of late-onset, steroid-resistant diarrhoea with features of steatorrhoea, prompting treatment with PERT, particularly where faecal elastase levels are low. Rare complications, such as hepatic steatosis, which was observed in our patient, require careful clinical investigation and suspicion as an isolated irAE, or at least as a complication of a pre-existing irAE in the context of ICI therapy, unless there is evidence to suggest otherwise.

With anti-PD-1 mAb therapy becoming the standard of care, a rise in the incidence of irAEs is anticipated, with a growing number of patients expected to present to multiple different medical specialties. This emphasises the ever-increasing importance of individual organ specialists working collaboratively as part of a multidisciplinary team in treating and managing patients early and effectively. Management of irAEs will improve with greater research and experience surrounding the use of current and new ICIs, and as such, there is scope for establishing and improving guidelines.
